# Sphingolipidomics of drug resistant *Candida auris* clinical isolates reveal distinct sphingolipid species signatures

**DOI:** 10.1016/j.bbalip.2020.158815

**Published:** 2021-01

**Authors:** Mohit Kumar, Ashutosh Singh, Sonam Kumari, Praveen Kumar, Mohd. Wasi, Alok K. Mondal, Shivaprakash M. Rudramurthy, Arunaloke Chakrabarti, Naseem A. Gaur, Neil A.R. Gow, Rajendra Prasad

**Affiliations:** a Amity Institute of Integrative Science and Health, and Amity Institute of Biotechnology, Amity University Gurgaon, Haryana, India; b Yeast Biofuel Group, International Centre for Genetic Engineering and Biotechnology, New Delhi, India; c Department of Biochemistry, University of Lucknow, Lucknow, India; d School of Life Sciences, Jawaharlal Nehru University, New Delhi, India; e The Postgraduate Institute of Medical Education and Research (PGIMER) Chandigarh, India; f Medical Research Council Centre for Medical Mycology, University of Exeter, Geoffrey Pope Building, Stocker Road, Exeter EX4 4QD, UK

**Keywords:** MDR, multidrug resistance, NAC, non-albicans *Candida*, FLC, fluconazole, AmB, amphotericin B, MS, mass spectrometry, SLs, sphingolipids, Liquid chromatography-tandem mass spectrometry, LC-MS/MS, FLC^R^, FLC-resistant, AmB^R^, AmB-resistant, FLC^R^ + AmB^R^, both FLC and AmB resistant, MIPC, mannosyl-inositol-phosphoceramides, M(IP)_2_C, mannosyl-diinositol-phosphoceramides, PDREs, Pdr1/Pdr3 response elements, PCA, principal component analysis, MYR, myriocin, AbA, aureobasidin A, SPT, serine palmitoyl-CoA transferase, IPC, inositolphosphorylceramide, GlcCer, glucosylceramide, DHS, dihydrosphingosine, SPH, sphingosine, S1P, sphingosine-1-phosphate, DHS1P, dihydrosphingosine-1-phosphate, PHS, phytosphingosine, PHS1P, phytosphingosine-1-phosphate, Glucosyl-SPH, glucosyl sphingosine, dhCer, dihydroceramide, Cer, ceramides, αOH-Cer, αhydroxy ceramides, PCer, phytoceramide, αOH-PCer, αhydroxy phytoceramide, αOH-GlcCer, αhydroxy glucosylceramide, IPCs, inositol phosphoryl ceramides, Multiple drug resistance, LC-MS/MS, Sphingolipids, Glucosylceramides, Phytoceramide

## Abstract

Independent studies from our group and others have provided evidence that sphingolipids (SLs) influence the antimycotic susceptibility of *Candida* species. We analyzed the molecular SL signatures of drug-resistant clinical isolates of *Candida auris*, which have emerged as a global threat over the last decade. This included Indian hospital isolates of *C. auris*, which were either resistant to fluconazole (FLC^R^) or amphotericin B (AmB^R^) or both drugs. Relative to *Candida glabrata* and *Candida albicans* strains, these *C. auris* isolates were susceptible to SL pathway inhibitors such as myriocin and aureobasidin A, suggesting that SL content may influence azole and AmB susceptibilities. Our analysis of SLs confirmed the presence of 140 SL species within nine major SL classes, namely the sphingoid bases, Cer, αOH-Cer, dhCer, PCer, αOH-PCer, αOH-GlcCer, GlcCer, and IPC. Other than for αOH-GlcCer, most of the SLs were found at higher concentrations in FLC^R^ isolates as compared to the AmB^R^ isolates. SLs were at intermediate levels in FLC^R^ + AmB^R^ isolates. The observed diversity of molecular species of SL classes based on fatty acyl composition was further reflected in their distinct specific imprint, suggesting their influence in drug resistance. Together, the presented data improves our understanding of the dynamics of SL structures, their synthesis, and link to the drug resistance in *C. auris*.

## Highlights

• *Candida auris* isolates are susceptible to sphingolipid inhibitors myriocin and aureobasidin A.
• The distribution of sphingolipid species is distinct among *C. auris* isolates resistant to different antifungals.
• Phytoceramides are the most abundant class of sphingolipid.
• Cer(d18:1/18:1) is the major of ceramide species in *C. auris*.
• d19:2 glucosylceramide backbone is typically in abundance in AmB resistant *C. auris* isolates.

## 1 Introduction

Increasing antimicrobial resistance in pathogenic fungi is becoming a global health threat and eroding our ability to control fungal infections with a limited armamentarium of antifungals [1]. Most of the fungal infections associated with significant mortality and antimicrobial resistance are triggered by opportunistic human fungal pathogens [1,2]. The major pathogens, *Candida albicans*, *Aspergillus fumigatus*, and *Cryptococcus neoformans*, may survive in anatomically distinct locations within the host and are capable of fostering deep-seated infections in patients with compromised immune systems [3]. In contrast to the common *C. albicans*, non-albicans *Candida* (NAC) species are evolving as problematic drug resistance pathogens [1]. The recent emergence of multiple drug-resistant *Candida auris* clades within a short period over five continents has become a global concern [[4], [5], [6]]. Each clade is largely clonal, spreading quickly in hospitals, and is persistent on surfaces and in bedding [2,6].

A multicenter study on three continents reported that ~93% of *C. auris* isolates were resistant to fluconazole (FLC), ~35% to amphotericin B (AmB), ~41% resistant to two antifungals (FLC and AmB) and ~4% resistant to all three major classes of antifungals (FLC, AmB, and echinocandins) (4). Azoles are front-line drugs in systemic infections against most *Candida* species that target the ergosterol biosynthesis pathway by inhibiting 14α-demethylase encoded by *ERG11* [7]. Notably, initial analyses of drug-resistant *C. auris* isolates identified well-characterized examples of azole resistant in *C. auris* [4,[8], [9], [10]]. Whole-genome sequencing (WGS) of azole resistant *C. auris* isolates confirmed the presence of mutations in azole target *ERG11* [4] and phenotypic studies demonstrated that overexpression of drug transporters genes could occur to the drug efflux pumps *CDR1*/*CDR2* and *MDR1* [8,10,11]. In several instances, *C. auris* isolates displayed resistance to polyenes that bind fungal membrane ergosterol [12,13]. The transcriptomic profile of *C. auris* with the treatment of AmB also revealed altered expression of sterol biosynthesis genes [9]. Despite the well-known mechanisms of azoles and polyenes resistance, our mechanistic understanding of antifungal resistance of *C. auris* is incomplete. It remains unclear why *C. auris* isolates can easily acquire resistance to multiple antifungals [14].

Several antifungal drugs target membrane ergosterol and enzymes involved in lipid biosynthesis, including sphingolipid (SLs), biosynthesis which are critical for many cell homeostatic processes [15,16]. These drugs target molecules derived from non-SL precursors (serine and palmitoyl Co-A), in the endoplasmic reticulum (ER), with eighteen-carbon amino-alcohol backbones [17]. A genome-wide genetic screen of *C. albicans* transposon-mediated mutant library revealed that the null mutants of *CaFEN1* or *CaFEN12* (encoding enzymes for the synthesis of very-long-chain fatty acids) became resistant to FLC [18]. Mass spectrometry (MS) analyses demonstrated changes in cellular SLs composition in both these mutants, including substantially increased levels of several mannosyl-inositol-phosphoceramides (MIPC) with shorter fatty-acyl chains [18]. *CaFEN1* and *CaFEN12* are also crucial for AmB susceptibility, cell wall integrity, hyphae, and biofilms formation in *C. albicans* [19]. The mannosyl-diinositol-phosphoceramides (M(IP)_2_C) depletion in *ipt1*Δ mutant in *S. cerevisiae* demonstrated increased susceptibility to plant defensins and decreased susceptibility to oxidative stress [20]. The glucosylceramide (GlcCer) synthesis mutant was observed to affect growth at different pHs in *C. neoformans* [21] and displayed increased susceptibility to sodium dodecyl sulfate (SDS) and FLC in *C. albicans* [22]. Furthermore, it has also been shown that the *ORM* gene that codes for SL regulatory proteins knockout mutants imposed ER stress responses and influenced azoles susceptibility in *A. fumigatus* [23]. Interestingly, ceramide synthase *LAC1*, *SUR2*, *LCB2*, and *IPT1* genes have Pdr1/Pdr3 response elements (PDREs) suggesting regulation by the Pdr1 transcription factor, which is a known regulator of azole resistance in several fungi [24].

Previous independent studies on *Candida* by our group and others demonstrated that lipids are tightly linked to MDR (Multiple Drug Resistance), and that membrane composition can influence the drug susceptibilities [25,26]. Large scale MS-based lipidomic studies have proven useful in establishing the links between specific lipid structures, their levels, and physiological functions [27,28]. For example, Singh et al., showed that mitochondrial lipids are associated with cell wall integrity and azole resistance in *C. albicans* [28]. A recent, multi-omics study between a FLC susceptible and a FLC resistant isolate of *C. auris*, has reported a semi-quantitative assessment of the lipidome [29]. This data described the relative changes between phosphoglycerides (PGL) and neutral lipids viz., diacylglycerols (DAG), triacylglycerols (TAG), and sterol intermediates [29]. The role of each lipid class, including SLs, may be equally crucial in *C. auris* and prime drug target to treat fungal infections and therefore demand attention. In this study, we focused on the characterization of the dynamics of SLs specific to recovered clinical drug resistance in *C. auris*. To assess the relevance of different sphingolipidomic signatures, we used three categories of clinical isolates of *C. auris*: (i) FLC-resistant (FLC^R^), (ii) AmB-resistant (AmB^R^) and (iii) both FLC and AmB resistant (FLC^R^ + AmB^R^). Our data revealed that all the *C. auris* strains in this study were susceptible to SL pathway inhibitor MYR, compared to *C. albicans* and *C. glabrata*. High-throughput liquid chromatography-tandem mass spectrometry (LC-MS/MS) based sphingolipidomics identified 140 SLs species in *C. auris* and defined the SL molecular species imprints specific to drug resistance. Our data provide insights into comprehensive SL landscape with some correlating with antifungal resistance in *C. auris*.

## 2 Materials and methods

### 2.1 Chemicals

Lipid standards were purchased from Sigma Aldrich, USA, and Avanti polar lipids Inc., USA. All solvents and chemicals, unless specifically mentioned, were LCMS (liquid chromatography-mass spectrometry) grade purchased from Sigma Aldrich, USA, and Fisher Scientific, UK.

### 2.2 Strains and culture conditions

*C. auris* strains were provided by National Culture Collection of Pathogenic Fungi (NCCPF), Indian Council of Medical Research (ICMR), New Delhi, sponsored National facility at the Mycology Division, Department of Medical Microbiology, Postgraduate Institute of Medical Education and Research (PIGMER), Chandigarh, India. *C. albicans* and *C. glabrata* strains used in this study were susceptible to FLC and AmB (Table 1). The laboratory strain *C. albicans* (SC5314/ATCC MYA-2876) was originally procured from ATTC (American Type Culture Collection, USA) and *C. glabrata* (BG2) was obtained from Centre for DNA Fingerprinting and Diagnostics, India. Cultures were maintained on YEPD agar plates at 30 °C. For lipid extraction, cells grown to an OD_600_ of 0.1 were inoculated in YEPD medium, then allowed to grow to OD_600_ 0.8 to 1. Approximately 5 × 10^8^ cells were harvested, washed with dH_2_O thrice before lipid extraction.

**Table 1:** Antifungal and sphingolipid inhibitors susceptibility profiling.

Strains	MIC50 (μg/mL)			
	AmB	FLC	MYR	ABA
NCCPF_470147	1	≥64	0.25	0.06
NCCPF_470026	1	≥64	0.12	0.03
NCCPF_470157	1	≥64	0.12	0.01
NCCPF_470055	1	32	0.06	0.01
NCCPF_470151	2	≥64	0.06	0.01
NCCPF_470114	4	≥64	0.5	0.03
NCCPF_470154	4	≥64	0.25	0.01
NCCPF_470140	4	1	0.12	0.06
NCCPF_470161	4	1	0.03	0.06
NCCPF_470097	2	1	0.12	0.01
CBS10913T	0.5	8	0.01	0.25
*C. albicans* SC5314	0.06	0.5	≥4	0.5
*C. glabrata* BG2	0.5	8	≥4	0.5

### 2.3 Drug susceptibility assessment

Minimum inhibitory concentrations (MIC) for FLC, AmB, against the *C. auris* strains, were determined by broth microdilution with two-fold serial dilutions in RPMI-1640 medium, as described in Clinical and Laboratory Standards Institute (CLSI, formerly NCCLS, USA) [30]. The growth kinetics was performed by a micro-cultivation method in a 96-well plate using Liquid Handling System (Tecan, Austria) in YEPD broth at 30 °C. Briefly, overnight grown yeast cultures were diluted to OD_600_ = 1.0, and 10 μL of each culture was mixed with 190 μL YEPD broth in 96 well plates, and OD_600_ was measured at every 30 min of an interval to up to 24 h. FLC (3 mg/mL) and AmB (2 mg/mL) and MYR (2.4 mg/mL) were dissolved in DMSO, AbA (1 mg/mL) was prepared in ethanol. *C. krusei* (ATCC 6258) and *C. parapsilosis* (ATCC 22019) were used as quality control reference strains.

### 2.4 Lipid extraction

Lipid extraction was performed as described earlier [31]. Briefly, about 5 × 10^8^ cells were suspended in 1.5 mL Mandala buffer (ethanol: dH_2_O: diethyl ether: pyridine: NH_4_OH (15:15:5:1:0.018; v/v)). To each sample 50 mg of glass beads (0.4–0.6 mm), C17 ceramide (d18:1/17:0; N-heptadecanoyl-D-erythro-sphingosine) and C17 sphingosine (C17 base; D-erythro-sphingosine) (as internal standards) were added and cells were then broken using Fast prep (MP biomedical). The samples were then kept at 60 °C for 1 h, with periodic vortexing and sonication at intervals of 15 min. Cell debris was removed by centrifugation (3000 rpm, 4 °C, 10 min) and the supernatant was dried using N_2_ gas. Next, Bligh and Dyer's extraction was performed by dissolving the pellet into chloroform: methanol in ratio 1:2 (v/v) and incubated at 37 °C for 1 h [32]. Phase separation was performed by adding 1 mL chloroform and 1 mL dH_2_O with vortexing. The hydrophobic layer was taken, dried using N_2_ gas. Further, mild alkaline hydrolysis was performed using 0.5 mL chloroform, and 0.5 mL 0.6 M methanolic KOH was added into the dry lipid samples, then vortexed and incubated at room temperature for 1 h. Then 0.325 mL 1 M HCl and 0.125 mL dH_2_O was added and vortexed. The hydrophobic layer was dried with N_2_ gas and stored at −20 °C until further use.

### 2.5 Mass spectrometry analysis

SLs were detected in base hydrolyzed lipid samples by multiple reaction monitoring (MRM) approaches described previously [31], using the QTRAP® 4500 (SCIEX, USA). Extracted lipids were suspended in a buffer containing 1 mM ammonium formate + 0.2% formic acid in methanol (Buffer B). 5 μl sample was delivered by Pump/Autosampler to the HPLC fitted with Kinetex® 1.7 μm C8 100 Å, 50 × 2.1 mm column (Phenomenex, USA). A two-buffer mobile system was used: 2 mM ammonium formate + 0.2% formic acid in H_2_O (Buffer A) and Buffer B. Quantification of SL species achieved by internal standard normalization method. The lipid data were further normalized to per O.D. cells, and after that, mol% calculated.

### 2.6 Statistics

Each analysis was performed in triplicates. Student's *t*-test was used to determine the statistical significance, and a *p*-value of ≤0.05 was considered significant. The SYSTAT software (Version 13.0) was used to perform the principal component analysis (PCA).

## 3 Results

### 3.1 Drug susceptibility profiles of *C. auris* clinical isolates

**Supplementary Fig. 1:**
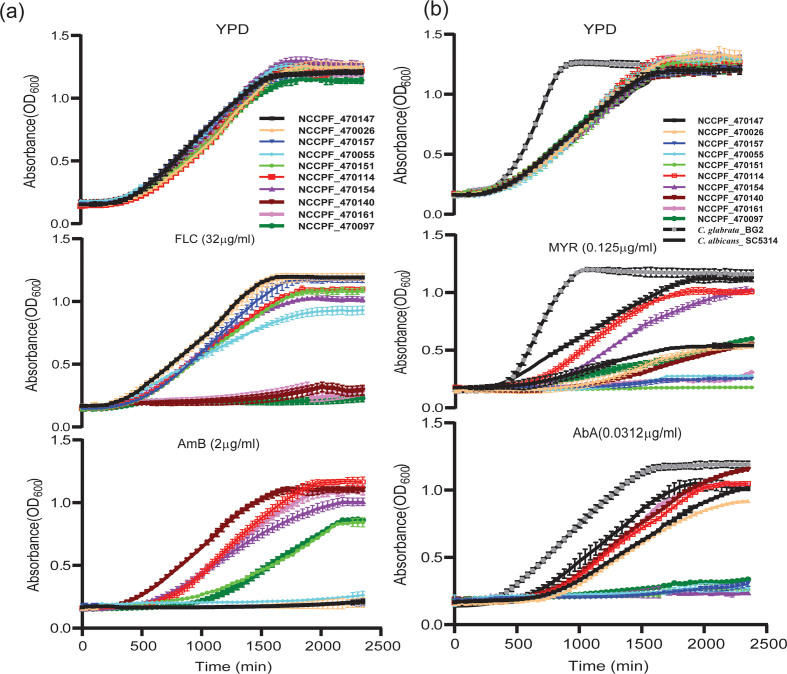
Growth kinetics of the isolates (a) in the presence of FLC and AmB (b) in the presence of MYR and AbA.

Breakpoints for resistance to antifungals agents in *C. auris* have not been formally established. Our findings are based on the breakpoint suggested by the Centers for Disease Control and Prevention (CDC, https:/www.cdc.gov/fungal/candida-auris/recommendations.html) by analyzing the data on related *Candida* spp. The MICs of all the isolates used in this study were measured against two antifungals, namely AmB and FLC. MIC values ≥ 32 μg/ml and ≥2 μg/ml for FLC and AmB respectively were considered as resistant. Our selection included four FLC^R^ isolates NCCPF_470147, NCCPF_470026 and NCCPF_470157, with MIC_50_ ≥ 64 μg/ml and NCCPF_470055 with MIC_50_ 32 μg/mL. Three AmB resistant (AmB^R^) clinical isolates were selected in which NCCPF_470140 and NCCPF_470161 have MIC_50_ at 4 μg/mL, and NCCPF_470097 have MIC_50_ at 2 μg/mL. These seven isolates did not display cross-resistance to other antifungals classes (data not shown). Three clinical isolates NCCPF_470154, NCCPF_470114, and NCCPF_470151 were also included in this study, with resistance to both FLC and AmB (FLC^R^ + AmB^R^) (MIC_50_ for FLC and AmB were ≥32 μg/mL and ≥2 μg/mL, respectively) (Table 1). Growth kinetics of the isolates on FLC and AmB also validated our MIC results (Supplementary Fig. 1a).

### 3.2 *C. auris* isolates are susceptible to SL synthesis inhibitors

Recent reports pointed out the role of SL in drug resistance, specifically against azole and polyenes. Therefore, the drug susceptibilities of select isolates were tested on SL inhibitors, namely myriocin (MYR) and aureobasidin A (AbA). Irrespective of resistance types, all the drug-resistant isolates were susceptible to MYR with MIC_50_ ranged between 0.01 and 0.5 μg/mL in comparison with *C. glabrata* (BG2) and *C. albicans* (SC5314) strains (MIC_50_ was ≥4 μg/ml) (Table 1). Isolate NCCPF_470114 (both FLC^R^ + AmB^R^) showed the maximum MIC_50_ 0.5 μg/mL for MYR, which was relatively high from other isolates. AbA MIC_50_ ranged from 0.06 to 0.25 μg/mL, which was also less than *C. albicans* (SC5314) and *C. glabrata* (BG2) (Table 1). Independent studies [19,33], including ours, reported that azole and AmB susceptible *C. albicans* SC5134 and *C. glabrata* BG2 present higher MIC_50_ values towards tested sphingolipid inhibitors. These strains highlighted the comparative higher susceptibility of *C. auris* strains towards SL inhibitors implying relevance of SLs in antifungal resistance. The growth kinetics of all tested *C. auris* isolates in the presence of SL inhibitors confirmed MIC data (Supplementary Fig. 1b).

### 3.3 *C. auris* isolates harbour all major SL classes

By employing high-throughput LCMS analysis in MRM mode with positive ion, we have detected and quantified all major SL classes and their species in *C. auris*. The identified SLs of *C. auris* belong to nine classes namely, sphingoid bases (or LCB's), dihydroceramide (dhCer), ceramides (Cer), αhydroxy ceramides (αOH-Cer), phytoceramide (PCer), αhydroxy phytoceramide (αOH-PCer), GlcCer, αhydroxy glucosylceramide (αOH-GlcCer) and inositol phosphoryl ceramides (IPCs). Further sphingoid bases (LCBs) were divided into seven subclasses namely, dihydrosphingosine (DHS), sphingosine (SPH), sphingosine-1-phosphate (S1P), dihydrosphingosine-1-phosphate (DHS1P), phytosphingosine (PHS), phytosphingosine-1-phosphate (PHS1P) and glucosyl sphingosine (Glucosyl-SPH). The total sphingoid bases were low in abundance, except for DHS, which exhibited relatively higher abundance (0.144 mol%). Moreover, the total sphingoid base amount did not show significant variation between FLC^R^, AmB^R^, and FLC^R^ + AmB^R^ isolates (Supplementary file 1, Fig. 1).

The utilization of sphingoid bases in the SL pathway results in the dhCer structures formed by an amide linkage with fatty acid [34]. In *C. auris*, dhCer was present at an average of 21.2 mol% (% of total SL), which significantly varies between the FLC^R^ and AmB^R^ isolates. For instance, the amount of dhCer was higher in FLC^R^ isolates with an average of 25.5 mol%, and, in comparison, a much lesser amount was present in AmB^R^ isolates (15.5 mol%) (Supplementary file 1, Fig. 1).

Among *C. auris* isolates, both the Cer and αOH-Cer classes followed a similar distribution pattern, as was the case with dhCer. Thus, Cer and αOH-Cer classes exhibited a higher amount in FLC^R^ than in the AmB^R^ isolates. The next intermediate of the SL biosynthetic pathway is PCer, which was found to be the most abundant biosynthetic intermediate in *C. auris*, representing 46 mol% of total SLs. However, its levels showed no significant variations in contents and remained unchanged between our tested drug-resistant isolates. Our analysis also detected α-hydroxylated PCer (αOH-PCer) with 5.6 mol% (%of total SL). In comparison with AmB^R^ isolates, αOH-PCer was significantly higher in FLC^R^ isolates. The major complex SLs in *C. auris* were represented by αOH-GlcCer, followed by GlcCer and IPCs. Among these, glycosylated SL, GlcCer was higher in FLC^R^ (2.7 mol%), while αOH-GlcCer was significantly higher in AmB^R^ isolates (25.2 mol%). IPCs are the third complex SL class found in our analysis, which did not exhibit any significant differences among these isolates. In general, except for αOH-GlcCer, most of the class of SLs were significantly higher in FLC^R^ isolates than in the AmB^R^. It is important to note here that the higher classes of IPCs, i.e., MIPC and M(IP)_2_C of complex SL, were below detection in our analysis in positive ion MS.

**Fig. 1:**
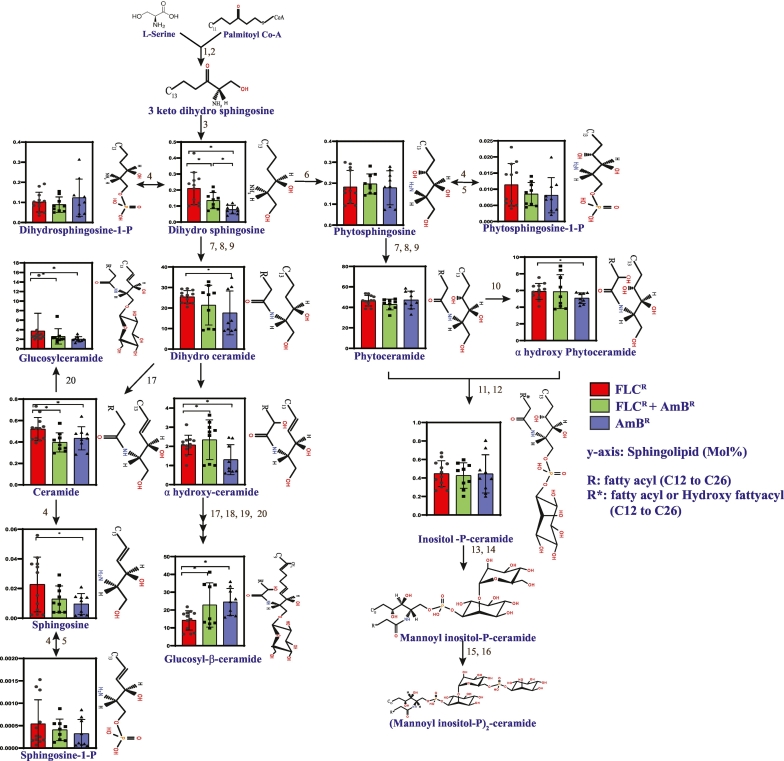
SL metabolism, its intermediates, and its total abundance within the three groups of *C. auris* clinical isolates. Based on the SL profile obtained from ten *C. auris* isolates, the total mol% of the identified SL classes was plotted as bar graph along the predicted pathway to highlight SL variations at different steps of the synthesis. The red, blue, and green bars represent mol% of SL intermediates in FLC^R^, AmB^R^, and FLC^R^ + AmB^R^, respectively. SL biosynthesis genes were retrieved from http://www.candidagenome.org/. The biosynthetic pathway is composed of the following enzymatic steps: serine palmitoyltransferase [1, 2], 3-ketodihydrosphingosine reductase [3], sphingosine kinase [4], long-chain base-1-phosphate phosphatase [5], sphinganine C4-hydroxylase [6], ceramide synthases [7,8,9], alpha-hydroxylase [10], IPC synthases [11, 12], MIPC synthase [13, 14], M(IP)_2_C synthase [15, 16], Δ4-desaturase [17], Δ8-desaturase [18], SL C9 methyltransferase [19] and GlcCer synthase [20]. The lipid structures depicted here have been adopted from www.lipidmaps.org.

### 3.4 Drug-resistant *C. auris* isolates display a distinct SL signature

In fungi, the SLs consist of the LCB backbone, which is linked to a fatty acid at the C2 position by an amide-linkage and has esterified polar head at the C1 position [35]. In our MS analysis, we identified a total of 140 SL species, based on fatty acid chain length and unsaturation. These included species with d18:0, d18:1, d18:2 d19:2, and t18:0 LCB backbones. Of note, LCB with d18:0 and t18:0 backbone constituted the major sphingoid species in *C. auris*. However, the other sphingoid backbones with d19:2 and d18:2 have also been detected, albeit much lower in number.

#### 3.4.1 SPH and DHS show significant variations in C. auris isolates

Sphingoid bases are long-chain aliphatic amino alcohols (LCB), which serve as precursors to a variety of SLs. SPH specifically refers to (2S,3R,4E)-2-amino-4-octadecen-1,3-diol, C18 aliphatic chain with an amine group in C2, C1, and C3 hydroxyl group, and C4 double bond, which forms the backbone structure of complex SLs. SPH content showed significant variation between AmB^R^ and FLC^R^ isolates and was higher in FLC^R^ as compared to AMB^R^ isolates. FLC^R^ NCCPF_470157 isolate had the highest SPH (0.038 mol%) level, while the least SPH level (0.006 mol%) was found in AmB^R^ NCCPF_470140 isolate. No significant differences in SPH contents were observed in the isolates resistant to both FLC^R^ and AMB^R^ antifungals. Phosphorylated sphingosine (S1P) had also been detected in our analysis but displayed no significant variation among the tested clinical isolates.

**Fig. 2:**
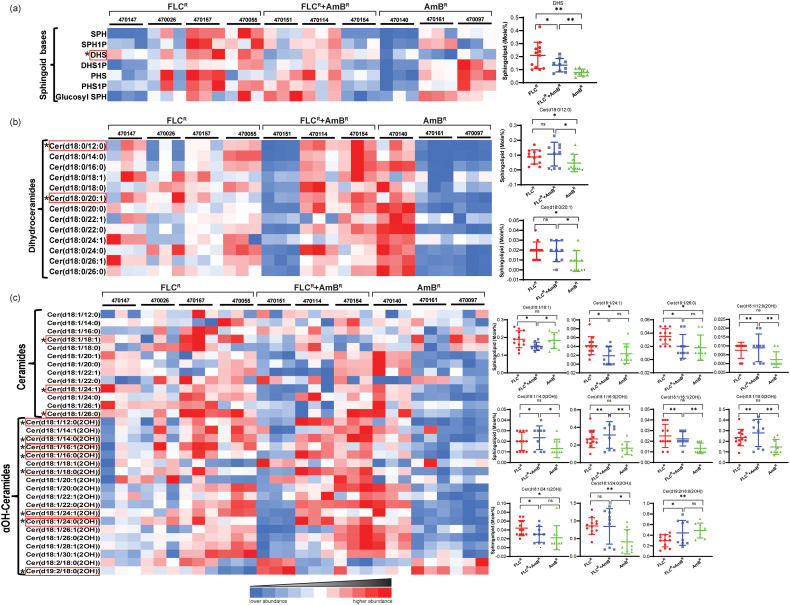
Comparative analysis of sphingoid bases, dhCer, Cer and αOH-Cer species in clinical isolates of *C. auris*: (a) 7 sphingoid bases were detected among which DHS showed significant differences among the three sets of isolates. (b) DhCer are abundant in *C. auris*. 13 species of dhCer were detected which were differentiated on the basis of fatty acid chain length. dhCer with fatty acyl chain length of 12:0 and 20:1 was displayed significant changes among the tested isolates. (c) Cer and αOH-Cer were detected in *C. auris*. 11 species among these show significant changes. The unusual sphingoid backbone d19:2 was detected in all isolates and particularly showed enrichment in AmB^R^. Data for all the replicates are shown in the colored diagram. Red boxed SL species distribution and its significances are highlighted in the right panels (distribution plots). The distribution plots were plotted using the individual value of each sample within the group and compared with the other groups. The data can be found in Supplementary File 1. ‘*’ and ‘**’ represent *p*-values <0.05 and 0.01, respectively. ‘ns’ stands for no significant change. Comparative analysis of sphingoid bases, dhCer, Cer and αOH-Cer species in clinical isolates of *C. auris*: (a) 7 sphingoid bases were detected among which DHS showed significant differences among the three sets of isolates. (b) DhCer are abundant in *C. auris*. 13 species of dhCer were detected which were differentiated on the basis of fatty acid chain length. dhCer with fatty acyl chain length of 12:0 and 20:1 was displayed significant changes among the tested isolates. (c) Cer and αOH-Cer were detected in *C. auris*. 11 species among these show significant changes. The unusual sphingoid backbone d19:2 was detected in all isolates and particularly showed enrichment in AmB^R^. Data for all the replicates are shown in the colored diagram. Red boxed SL species distribution and its significances are highlighted in the right panels (distribution plots). The distribution plots were plotted using the individual value of each sample within the group and compared with the other groups. The data can be found in Supplementary File 1. ‘*’ and ‘**’ represent *p*-values <0.05 and 0.01, respectively. ‘ns’ stands for no significant change.

The variations in the DHS level were statistically significant and were a distinguishing feature between all three sets of isolates. For instance, in general, DHS amount was highest in all tested FLC^R^ isolates (0.28 mol%) and lowest in all AmB^R^ isolates (0.083 mol%) (Fig. 2a); however, showed intermediate levels (0.14 mol%) in the isolates, which were resistant to both the antifungals. As a specific example, the highest quantity of DHS was in FLC^R^ NCCPF_470157, and the lowest in AmB^R^ NCCPF_470161 (Supplementary file 1). In contrast, PHS levels, which is the second most abundant sphingoid base, did not show any dissimilarity among the different sets of isolates. Further, DHS1P, PHS1P, and Glucosyl SPH were either very low in content or insignificantly changed in isolates of *C. auris* (Fig. 2a).

#### 3.4.2 dhCer distribution among C. auris isolates reveals an abundance of Cer(d18:0/18:0) species

The 13 identified molecular species of dhCer, differed from each other based on the length of the fatty acid added from the long-chain fatty acid (LCFA) and very-long-chain fatty acid (VLCFA) synthesis arm of the SL pathway. The fatty acid chain length of dhCer in *C. auris* isolates varied in the carbon chain length from 12C to 26C, while the sphingoid backbone (d18:0) was the common denominator of all the species of dhCer. Among these, the dhCer with C18:0 fatty acid (stearic acid) was the most abundant species showing a relatively higher level in FLC^R^ isolates (9.7 mol%) as compared to AmB^R^ (7.7 mol%). Interestingly, dhCer with 24C was particularly higher in FLC^R^ isolates (9.53 mol%) as compared to AmB^R^ isolates (5.02 mol%). Among the three AmB^R^ isolates, NCCPF_470140 showed the highest amount (10.9 mol%) of Cer(d18:0/18:0) (Fig. 2b, Supplementary file 1). The Cer(d18:0/22:1) was the least abundant species of dhCer in *C. auris* isolates. Other dhCer species Cer(d18:0/12:0) and Cer(d18:0/20:1) also displayed a significant difference between the FLC^R^ vs. AmB^R^ and AmB^R^ vs. FLC^R^ + AmB^R^ isolates. The mol% distinction was also evident for Cer(d18:0/14:0), Cer(d18:0/18:1) and Cer(d18:0/26:0) species between FLC^R^ and AmB^R^ isolates (Fig. 2b).

#### 3.4.3 Cer(d18:1/18:1) and Cer(d19:2/18:0(2OH)) are the key Cer structures

Cer, a bioactive molecule that participates in various physiological processes, is produced by the desaturation of dhCer, and insertion of a double bond into the sphingoid backbone [36]. Our analysis could differentiate Cer molecular species based on the carbon chain length of fatty acid with the sphingoid backbone of d18:1 and identified a total of 13 species. Cer with the d18:1 backbone with 18:1 fatty acid (oleic acid), Cer(d18:1/18:1), was most abundant (0.185 mol%). There was no significant variation in fatty acid content between FLC^R^ and AmB^R,^ but there was a difference between FLC^R^ vs. FLC^R^ + AmB^R^ and between AmB^R^ vs. FLC^R^ + AmB^R^ isolates. Cer with C14:0, C16:0, C20:1, C20:0, C22:0 and C22:1, fatty acids were low in abundance in *C. auris*, and Cer(d18:1/12:0) was either not detected or not present in these isolates. Cer with VLCFA (C24:1, C24:0 and C 26:0) exhibited a significant variance within FLC^R,^ and AmB^R^ isolates where all these three Cer structures were abundant in FLC^R^ and scarce in AmB^R^ (Fig. 2c). The NCCPF_470157 (FLC^R^) isolate was particularly rich in Cer content as compared to all the isolates while the NCCPF_470151 and NCCPF_470161 isolates showed lowest Cer content (Supplementary file 1).

Our analysis also targeted 17 species of αOH-Cer with different hydroxylated fatty acids and two with different sphingoid backbone in *C. auris* clinical isolates. αOH-Cer with 24C fatty acid, Cer(d18:1/24:0(2OH)), was the most abundant species and showed significant variance between FLC^R^ and AmB^R^ isolates (Fig. 2). Interestingly, the αOH-Cer with a sphingoid backbone of d19:2 was present in a higher amount in all AmB^R^ isolates. NCCPF_470154 (FLC^R^ + AmB^R^) had the highest αOH-Cer content (3.5 mol%), and NCCPF_470097 (AmB^R)^ had the lowest αOH-Cer content (1.25 mol%). αOH-Cer molecular species contents with hydroxy fatty acid C12:0(2OH), C14:0(2OH), C16:0(2OH), C16:1(2OH), C18:0(2OH), and C24:0(2OH) varied significantly (Fig. 2c).

#### 3.4.4 PCer is an abundant SL in *C. auris*

**Fig. 3:**
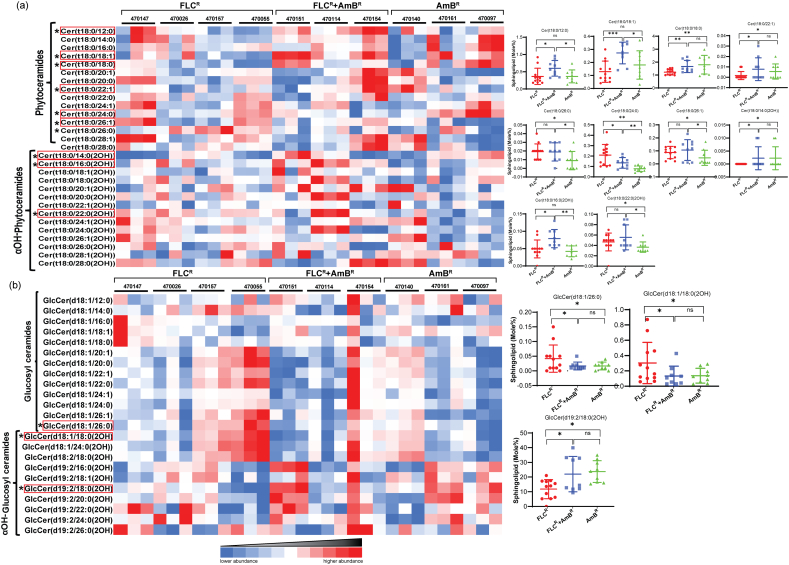
PCer, αOH-PCer GlcCer and αOH-GlcCer species in clinical isolates of *C. auris*: (a) PCer and αOH-PCer are enriched SL species in *C. auris*. Cer(t18:0/24:0) is the most abundant PCer and among all tested species of SLs. (b) GlcCer and αOH-GlcCer species distribution in *C. auris*. Data for all the replicates are shown in the colored diagram. The d19:2 sphingoid backbone was most abundant among the glycosylated SLs. Red boxed SLs species distribution and its significances are highlighted in the right panels (distribution plots). The distribution plots were plotted using the individual value of each sample within the group and compared with the other groups. The data can be found in Supplementary File 1. ‘*’ and ‘**’ represent *p*-values <0.05 and 0.01, respectively. ‘ns’ stands for no significant change. PCer, αOH-PCer GlcCer and αOH-GlcCer species in clinical isolates of *C. auris*: (a) PCer and αOH-PCer are enriched SL species in *C. auris*. Cer(t18:0/24:0) is the most abundant PCer and among all tested species of SLs. (b) GlcCer and αOH-GlcCer species distribution in *C. auris*. Data for all the replicates are shown in the colored diagram. The d19:2 sphingoid backbone was most abundant among the glycosylated SLs. Red boxed SLs species distribution and its significances are highlighted in the right panels (distribution plots). The distribution plots were plotted using the individual value of each sample within the group and compared with the other groups. The data can be found in Supplementary File 1. ‘*’ and ‘**’ represent *p*-values <0.05 and 0.01, respectively. ‘ns’ stands for no significant change.

Our analysis of SLs showed that PCer was the major SL in *C. auris*, wherein we identified 15 species based on fatty acid chain lengths of C12 to C28 with t18:0 sphingoid backbone. Among molecular species, PCer, with fatty acid C24:0 (lignoceric acid), was most abundant, followed by C26:0 (Cerotic acid). The distribution of Cer(t18:0/24:0) among resistant isolates showed its higher content in AmB^R^ isolate (26.7 mol%) and significantly lower in FLC^R^ (21.3 mol%) isolates. The second most abundant species Cer(t18:0/26:0), displayed different distribution among these isolates (Fig. 3a). PCer with C18:0 and C28:0 were also identified, but only Cer(t18:0/18:0) showed any significant difference between the FLC^R^ (1.2 mol%) and AmB^R^ (1.8% mol%) isolates. Other species with C12:0, C18:1, and C24:1 fatty acyls showed a significant disparity between at least two selected sets of isolates, while others showed strain-specific variations that could not be grouped. *C. auris* isolate, which was most abundant in PCer, was NCCPF_470097 with 53.8 mol%, while NCCPF_470026 was with the least amount of PCer with an average of 42 mol% (Supplementary file 1).

Our analysis could also detect 14 species of αOH-PCers with varying fatty acid chain lengths; however, were similar to the fatty acyl chains observed in case of PCers. The most abundant species of αOH-PCers was Cer(t18:0/24:0(2OH)), and this species displayed the significant difference between FLC^R^ and AmB^R^ isolates, higher mol% in FLC^R^ (5.19 mol%) as compared to AmB^R^ (4.48 mol%). Cer(t18:0/26:0(2OH)) was the other abundant species but showed no significant variation among the isolates (Supplementary file 1). Unlike the PCer, only a few species (14:0(2OH), 16:0(2OH), and 22:0(2OH)) of αOH-PCers showed significant variations in the levels within the isolates (Fig. 3a).

#### 3.4.5 GlcCer biosynthetic branch is active in *C. auris*

Our sphingolipidomic analysis revealed the presence of glycosyl derivatives as major complex SLs in *C. auris*, implying that the GlcCer branch of the SL pathway is active in *C. auris*. GlcCer formation requires the transfer of glucosyl-group of UDP-glucose to the αOH-Cer C1 hydroxyl group [31]. The most abundant GlcCer species was with C16:0 fatty acid (palmitic acid), which did not show any change between FLC^R^ and AmB^R^ isolates; however, it displayed a significant change in concentrations between AmB^R^ (0.75 mol%) and FLC^R^ + AmB^R^ (1.29 mol%) isolates. GlcCer species with VLCFA (C24:0, C26:0, and C26:1) were most variable in terms of mol% between the FLC^R^ and AmB^R^ isolates (Fig. 3b).

**Fig. 4:**
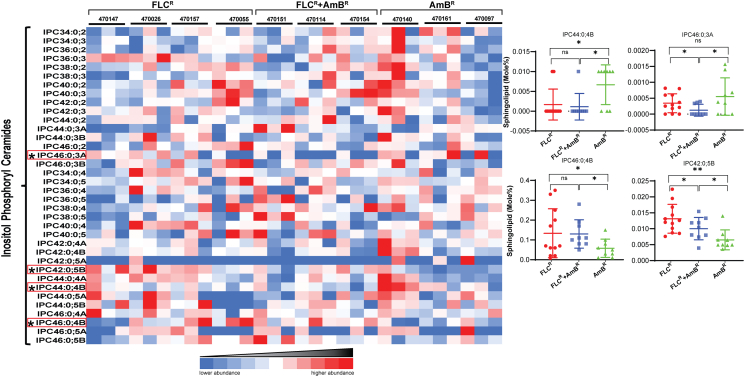
IPC species in clinical isolates of *C. auris*. Among the 36 targeted IPC species in our analysis, IPC44:0:4B, IPC46:0:3B, IPC46:0:4B and IPC42:0:5B show significant changes among the tested isolate groups. IPC species are represented as “total number of carbons in the sphingoid base and acyl chains: total number of carbon-carbon double bonds in the sphingoid base and acyl chains- number of hydroxyl groups present in the sphingoid base and acyl chains”. Data for all the replicates are shown in the colored diagram. Red boxed SLs species distribution and its significances are highlighted in the right panels (distribution plots). The distribution plots were plotted using the individual value of each sample within the group and compared with the other groups. The data can be found in Supplementary File 1. ‘*’ and ‘**’ represent *p*-values <0.05 and 0.01 respectively. ‘ns’ stands for no significant change. IPC species in clinical isolates of *C. auris*. Among the 36 targeted IPC species in our analysis, IPC44:0:4B, IPC46:0:3B, IPC46:0:4B and IPC42:0:5B show significant changes among the tested isolate groups. IPC species are represented as “total number of carbons in the sphingoid base and acyl chains: total number of carbon-carbon double bonds in the sphingoid base and acyl chains- number of hydroxyl groups present in the sphingoid base and acyl chains”. Data for all the replicates are shown in the colored diagram. Red boxed SLs species distribution and its significances are highlighted in the right panels (distribution plots). The distribution plots were plotted using the individual value of each sample within the group and compared with the other groups. The data can be found in Supplementary File 1. ‘*’ and ‘**’ represent *p*-values <0.05 and 0.01 respectively. ‘ns’ stands for no significant change.

Δ4-αOH-Cer with sphingoid backbone d18:1 undergoes Δ8-desaturation and result in the formation of Δ4, Δ8-Cer with d18:2 sphingoid backbone. Further, the addition of a methyl group at the C9 position of Δ4, Δ8-Cer forms 9-methyl-Δ4, Δ8-Cer with d19:2 sphingoid backbone of αOH-GlcCer. αOH-GlcCer was the major complex SLs in *C. auris* with the sphingoid backbone of d19:2, while αOH-GlcCer with sphingoid backbone of d18:1 and d18:2 were also detected. αOH-GlcCer species, GlcCer(d19:2/18:0(2OH)), with a hydroxylated fatty acid of 18C, was the most abundant species. This species showed a higher level in AmB^R^ (24.2 mol%) than in the FLC^R^ (13.3 mol%) isolates and also exhibited a significant difference between FLC^R^ and FLC^R^ + AmB^R^ isolates (Supplementary file 1). The second most plentiful GlcCer species detected were d18:1/18:0(2OH) and d19:2/16:0(2OH), wherein only the former showed the opposite trend as shown by d19:2/18:0(2OH). Altogether, AmB^R^ isolates showed ~2-fold higher amounts of αOH-GlcCer than the FLC^R^ isolates (Fig. 3b).

#### 3.4.6 Inositol phosphoryl ceramides have low abundance in *C. auris*

The total amount of IPCs with 36 species was unchanged in all tested isolates. However, IPC44:0;3A, IPC46:0;3A, IPC42:0;5B, IPC44:0;4B and IPC46:0;4B showed significant difference between at least two sets of isolates (Fig. 4). The most plentiful IPC species was IPC42:0;4B followed by IPC34:0;2 in *C. auris*. NCCPF_470140 (AmB^R^) isolates had a higher abundance of IPCs among tested *C. auris* isolates, while NCCPF_47014 isolate recorded the least amount of IPCs with 0.27 mol% (Supplementary file 1).

### 3.5 Principal component analysis (PCA) validates the statistically significant SL species differences

**Fig. 5:**
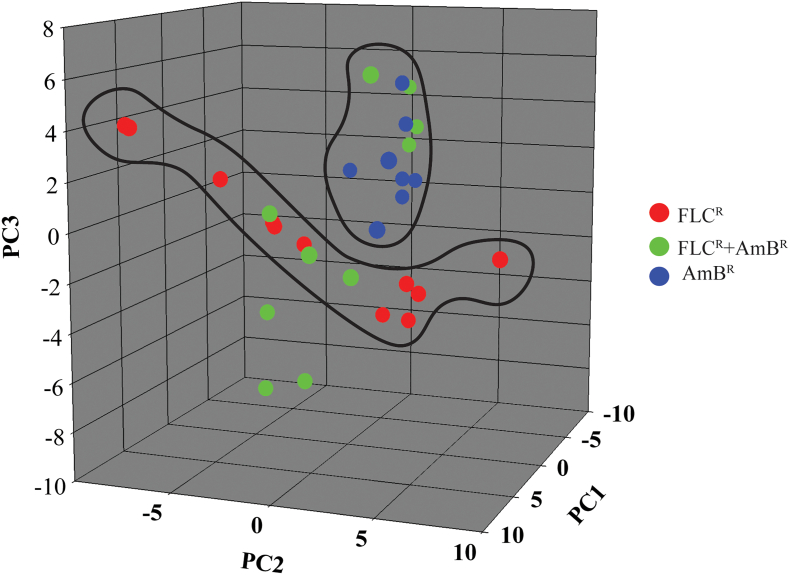
PCA analysis of SL molecular species datasets. PCA analyses of SLs showed the three replicates of each isolates sets are grouped together. The three groups of isolates FLC^R^, FLC^R^ + AmB^R^ and AmB^R^ are represented in red, green and blue colors respectively. The analysis showed that FLC^R^ and AmB^R^ isolates grouped separately, however, isolates with FLC^R^ + AmB^R^ dispersed with the FLC^R^ and AmB^R^. PCA was performed using the SYSTAT software (Version 13.0). PC1 (x-axis), PC2 (y-axis) and PC3 (z-axis) represented the three most variable principal components. The data for the loading values associated with each principal component can be found in Supplementary File 2. PCA analysis of SL molecular species datasets. PCA analyses of SLs showed the three replicates of each isolates sets are grouped together. The three groups of isolates FLC^R^, FLC^R^ + AmB^R^ and AmB^R^ are represented in red, green and blue colors respectively. The analysis showed that FLC^R^ and AmB^R^ isolates grouped separately, however, isolates with FLC^R^ + AmB^R^ dispersed with the FLC^R^ and AmB^R^. PCA was performed using the SYSTAT software (Version 13.0). PC1 (x-axis), PC2 (y-axis) and PC3 (z-axis) represented the three most variable principal components. The data for the loading values associated with each principal component can be found in Supplementary File 2.

PCA was performed using the mol% (% of total SLs) of the SL molecular species datasets between FLC^R^, FLC^R^ + AmB^R^ and AmB^R^ *C. auris* groups, to highlight the statistically significant SL variations among them. The analysis extracted 22 PCAs, of which the first three components showed maximum variations, scores which are depicted in (Fig. 5). We can see a marked separation of AmB^R^ and FLC^R^ groups. The observed overlap of AmB^R^ + FLC^R^ group over the AmB^R^ and FLC^R^ groups suggests a mixed distribution of lipid species that carry the marker for both FLC and AmB resistance. The loading values of the SL species responsible for their assignment to PCA 1, 2, and 3 can be found in Supplementary file 2. A close examination of the loading values of PCA1 show that Cer and GlcCer species with d19:2 backbone and α-hydroxylated fatty acyl (for example Cer(d19:2/18:0(2OH)), GlcCer(19:2/18:0(2OH)), etc.) are least abundant in FLC^R^ isolates. On the other hand, αOH-Cer (d18:1 backbone; 14–26 carbon fatty acyls) and dhCer (d18:0 backbone; 20–28 carbon fatty acyls) are abundant in FLC^R,^ and AmB^R^ + FLC^R^ isolates and significantly lower in AmB^R^ isolates.

Loading values of PCA 2 revealed that the sphingoid bases (namely SPH, SPH1P, DHS, DHS1P, and PHS1P), IPC structures (36:0;3, 44:0;4A, 44:0;5A, 46:0;2), were lower in abundance in AmB^R^ + FLC^R^ and AmB^R^ isolates. SL species IPC46:0;4B, Cer(d18:1/16:1(2OH)), Cer(t18:0/28:1) and GlcCer(d18:1/16:0) were particularly lower in content in AmB^R^ isolates, while IPC44:0;4B and Cer(d18:0/24:1) were lower in content only in AmB^R^ + FLC^R^ isolates. Likewise, the loading values of PCA 3 showed that αOH-Cer (d18:1 backbone; 16–24 carbon fatty acyls) and αOH-PCer (t18:0 backbone; 16–24 carbon fatty acyls) were significantly lower in AmB^R^ isolates while abundant in AmB^R^ + FLC^R^ isolates. Several GlcCer species (d18:1 backbone; 14–26 carbon fatty acyls) were significantly lower in AmB^R^ + FLC^R,^ and AmB^R^ isolates, while abundant in FLC^R^ isolates. Together, the PCA analysis identified specific SL species characteristics to each of the isolate groups and also validated the statistical significance of these datasets (Supplementary file 1, Supplementary file 2).

### 3.6 Comparative SL profile of drug-susceptible and resistant isolates

To identify the specific SL species variations that could be associated with drug resistance, we compared the SL profile of a drug-susceptible isolate (CBS10913T) with the various drug-resistant isolates. Our analysis revealed that drug-susceptible isolate (CBS10913T) has a distinct sphingolipid profile as compared to resistant isolates. For instance, the sphingoid bases (like S1P and PHS1P), dhCer and Cer (18C and 20C FA containing), αOH-Cer (16C and 30C FA containing) and PCer (28C FA containing), αOH-PCer (28C FA containing), IPC species, among others, were in abundance in CBS10913T strain (Fig. 7). On the other hand, PCer (14C to 24C FA containing), αOH-PCer (16C to 24C FA containing), among others, were present in low abundance in CBS10913T strain, compared to the resistant isolates (Fig. 7).

It is important to point out that while some of the SL species were present in higher amounts in *C. auris* isolates than those depicted in Fig. 7 since these species distributions between CBS10913T strain and the drug-resistant isolates was not statistically significant. For example, GlcCer(d19:2/18:0(2OH)) and Cer(t18:0/26:0) are abundant SL structures in CBS10913T strain, yet it did not show significant variations when compared to the resistant isolates. A comparison of the amounts of most abundant SLs present in different groups is represented in Supplementary Table 1.

## 4 Discussion

The available knowledge of the drug resistance mechanisms is unable to explain, global emergence of highly drug resistance *C. auris* as a new NAC spp. Notably, *C. auris* clinical isolates display raised resistance to FLC, but many also show collateral resistance to AmB or echinocandins [31,37]. With this increasing incidence of resistance, the scenario demands to look into possibilities of additional strategies that effect antifungal therapy. Most of the antifungals target the lipid metabolic pathway due to their uniqueness in fungi [38]. With the proven success rates in targeting the SLs, these metabolic pathways remain prime fungus-specific drug targets for antifungal drug discovery and development strategies [22]. SLs serve as signalling molecules and play a crucial role in cellular metabolic processes, organization of biomembranes, and in regulating fungal pathogenicity [23,48].

Notwithstanding, several independent studies on SL composition and their diverse roles in other yeasts and fungi, SL biosynthetic pathways in *C. auris* remain concealed. Apart from a single multi-omics study in *C. auris* that led to the identification of a few SLs species [29], this aspect remains obscure. Hence, a proper structural and composition analysis of SLs in *C. auris* is demanded to unravel the relevance of these lipids in the impressively higher observed resistance in this pathogen. In the present study, we focused on high throughput ESI-MS/MS analysis of SLs extracted from the ten drug-resistant clinical isolates *C. auris*. These isolates based on their single or collateral drug susceptibility to antifungals were clustered into three groups. In our analysis, we could identify a total of 154 SL species belonging to nine SL classes based on their structures. Detailed analysis of these SL classes unraveled the characteristics of SL species present in *C. auris*, starting from simple sphingoid base to complex SLs such as GlcCer and IPC.

**Fig. 6:**
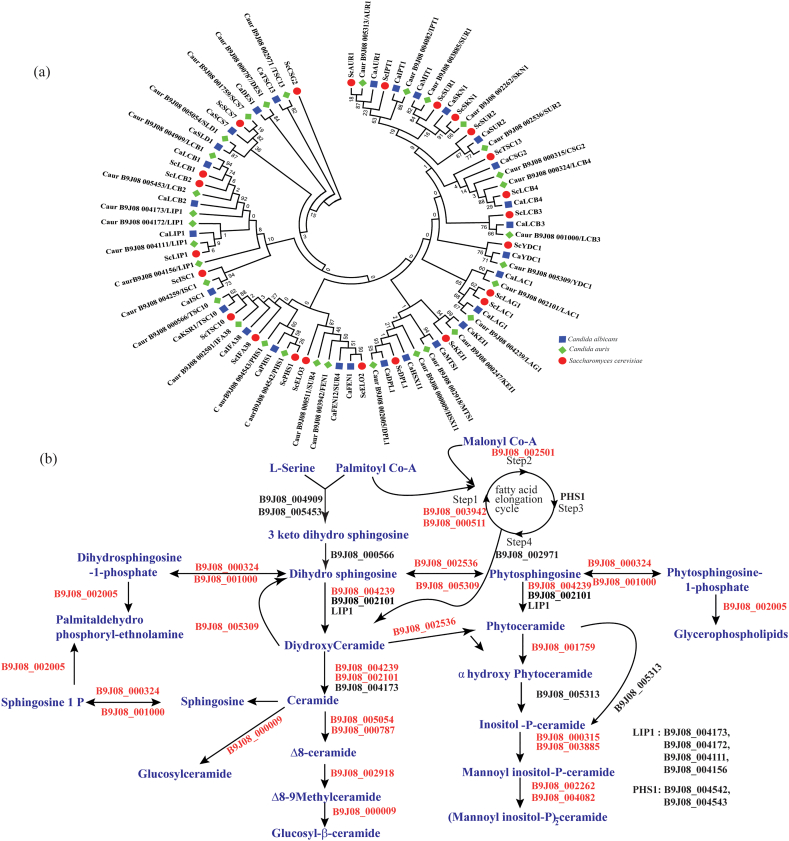
Phylogenetic analysis and predicted SLs pathway genes of *C. auris*. (a) Phylogenetic analysis of *C. auris* SL proteins displayed closeness to the SL protein of *C. albicans* than *S. cerevisiae*. Protein sequences of *C. auris*, *C. albicans*, and *S. cerevisiae* SL genes were retrieved from the *Candida* genome database (http://www.candidagenome.org/) and *Saccharomyces* genome database (https://www.yeastgenome.org/) respectively. Protein sequences were aligned by using ClustalW and phylogeny was constructed by MEGA6 with the Jones-Taylor-Thornton (JTT) model, and 1000 bootstrap replicates. (b) Based on homology studies with *C. albicans* and *S. cerevisiae*, genes coding for enzymes in the SL metabolic pathway of C. auris have been retrieved from the *Candida* genome database (CGD) and depicted in the pathway. The function of the genes was based on the orthology of genes in *C. albicans* and *S. cerevisiae*. The complete list of identified genes can be found in Supplementary File 3. Phylogenetic analysis and predicted SLs pathway genes of *C. auris*. (a) Phylogenetic analysis of *C. auris* SL proteins displayed closeness to the SL protein of *C. albicans* than *S. cerevisiae*. Protein sequences of *C. auris*, *C. albicans*, and *S. cerevisiae* SL genes were retrieved from the *Candida* genome database (http://www.candidagenome.org/) and *Saccharomyces* genome database (https://www.yeastgenome.org/) respectively. Protein sequences were aligned by using ClustalW and phylogeny was constructed by MEGA6 with the Jones-Taylor-Thornton (JTT) model, and 1000 bootstrap replicates. (b) Based on homology studies with *C. albicans* and *S. cerevisiae*, genes coding for enzymes in the SL metabolic pathway of C. auris have been retrieved from the *Candida* genome database (CGD) and depicted in the pathway. The function of the genes was based on the orthology of genes in *C. albicans* and *S. cerevisiae*. The complete list of identified genes can be found in Supplementary File 3.

Our analysis could quantify all primary SL biosynthetic intermediates and recorded their quantitative variations between AmB^R^, FLC^R,^ and AmB^R^ + FLC^R^ isolates. Many pathways of lipid biosynthesis and metabolism are conserved across the species ranging from yeast to human. Nevertheless, a notable difference exists in the SLs pathway of yeasts and mammals [15]. Based on the molecular SL species identification and gene homology, we predicted that the SL pathway in *C. auris* (Fig. 1, Fig. 6). Based on the close homology of SL genes with *C. albicans* and *S. cerevisiae* and present sphingolipidomics analysis, we could identify and predict 28 SL genes that play a role in the biosynthetic pathway in *C. auris* (Fig. 1, Fig. 6). Our analysis confirmed that in *C. auris*, similar to several *Candida* spp. both branches of complex SLs, leading to the formation of GlcCer and IPCs, are active (Fig. 1) [35,39]. This is distinct from SL metabolism in *S. cerevisiae* [40] and *C. glabrata* [41], where the IPC branch predominates (our unpublished data).

Among the identified nine classes, sphingoid bases are the simplest form of SLs intermediates. DHS (18:0) was the most common, and this exhibited significant variation in mol% distribution in different strains. For example, FLC^R^ resistant isolates showed higher levels of DHS as compared to AmB^R^ resistant isolates. Notably, DHS levels were also reported to be significantly higher in CRS-MIS (caspofungin reduced susceptibility - micafungin increased susceptibility) mutants in *A. nidulans* and *C. albicans* [41]. DHS transport mediated by *RTA2* was shown to be linked with FLC resistance in *C. albicans* [42]. Together, these studies suggest that DHS levels; influence drug susceptibilities in pathogenic fungi [41].

DHS has two fates. It can be hydroxylated at the C4 position into PHS (sphingoid base) by B9J08_002536/SUR2 or can be converted to Cer by Cer synthases encoded by B9J08_004239/LAG1, B9J08_002101/LAC1, and B9J08_004173/LIP1 (Fig. 6). Among these two intermediates, PHS is associated with AmB susceptibility, particularly in *FEN1* and *SUR1* mutants of *C. albicans* [19]. Our data also demonstrated that unlike in *Cryptococcus*, where Cer synthase preferably used 18:0 fatty acyl [31], *C. auris* primarily uses 18:1 fatty acyl as substrate. However, fatty acyls of 16:0, or 24:0 or 26:0 to form dhCer structures, were also detected. dhCer notably displayed enrichment in FLC^R^ isolates than the AmB^R^. PHS gets converted into PCer by the Cer synthase gene. For the synthesis of PCer, Cer synthases predominantly used C24:0 fatty acyl carbon followed by C26:0, C28:0, and C18:0. PCer was the most abundant SL species found in our analysis. Hydroxylation at the C2 position of PCer by B9J08_001759/*SCS7* results in conversion of PCer into αOH-PCer. The final production of SL biosynthesis is M(IP)_2_C, which is generated in three irreversible steps, mediated by enzymes B9J08_005313/*IPC1*, B9J08_003885/*SUR1*, B9J08_000315/*CSG2*, B9J08_004082/*IPT1*, and B9J08_002262/*SKN1*. Interestingly, the *IPT1* gene displays 57.42% sequence identity with the *S. cerevisiae* counterpart and 45.4% with *C. albicans* (Supplementary file 3). Our analysis with MRM in positive ion scanning mode was unable to detect the products of MIPC synthase and *IPT1* (M(IP)_2_C). More focused MS analysis using the negative ion approach is needed to analyze IPCs higher derivatives.

The analysis of GlcCer biosynthesis structures revealed the enzymes involved in this process; however, they remain uncharacterized. The second branch (Cer branch) of the SL pathway culminates at the formation of GlcCer by enzymes encoded by B9J08_005054/*SLD1*, B9J08_000787/*DES1*, B9J08_002918/*MTS1*, and B9J08_000009/*HSX11*. First, LCB undergoes Δ^4^-desaturation by a desaturase (B9J08_000787/*DES1*) and form d18:1 Cer backbone (Fig. 1). A desaturation and methylation that follow generate d19:2 backbone of ceramides. This extended pathway is not present in *S. cerevisiae*, but is present in *C. albicans* [39,43]. Cer structures consisting of non-hydroxylated fatty acyl, do not make a significant contribution to the GlcCer content of *C. auris* because (d19:2/18:0(2OH)) is the main GlcCer species in *C auris*, also reported in *Cryptococcus* [31].

The significant dissimilarity of dhCer, Cer, and αOH-Cer in drug-resistant isolates point to their possible influence on observed drug selective resistance in *C. auris*. We suggest that resistance to a specific antifungal could be linked to the amount and molecular species of SL. For example, in αOH-Cer class, a different sphingoid backbone (Cer(d19:2/18:0(2OH))) is rich in AmB^R^ strains as compared to other isolates. αOH-GlcCer was the major complex SLs present in all isolates, and its levels were raised in AmB^R^ isolates. SL pathway inhibitors such as *N*′-(3-bromo-4-hydroxybenzylidene)-2-methylbenzohydrazide (BHBM) and 3-bromo-*N*′-(3-bromo-4-hydroxybenzylidene) benzohydrazide (D0) highly synergistic when combined with FLC and AmB, suggesting a strong link between SL contents and drug resistance mechanism [44]. Based on our analysis and identification of biosynthetic intermediates, we have confirmed the major steps of SLs biosynthesis in *C. auris*, and confirmed the PCA based differences among different isolates (Fig. 5, Supplementary file 2).

**Fig. 7:**
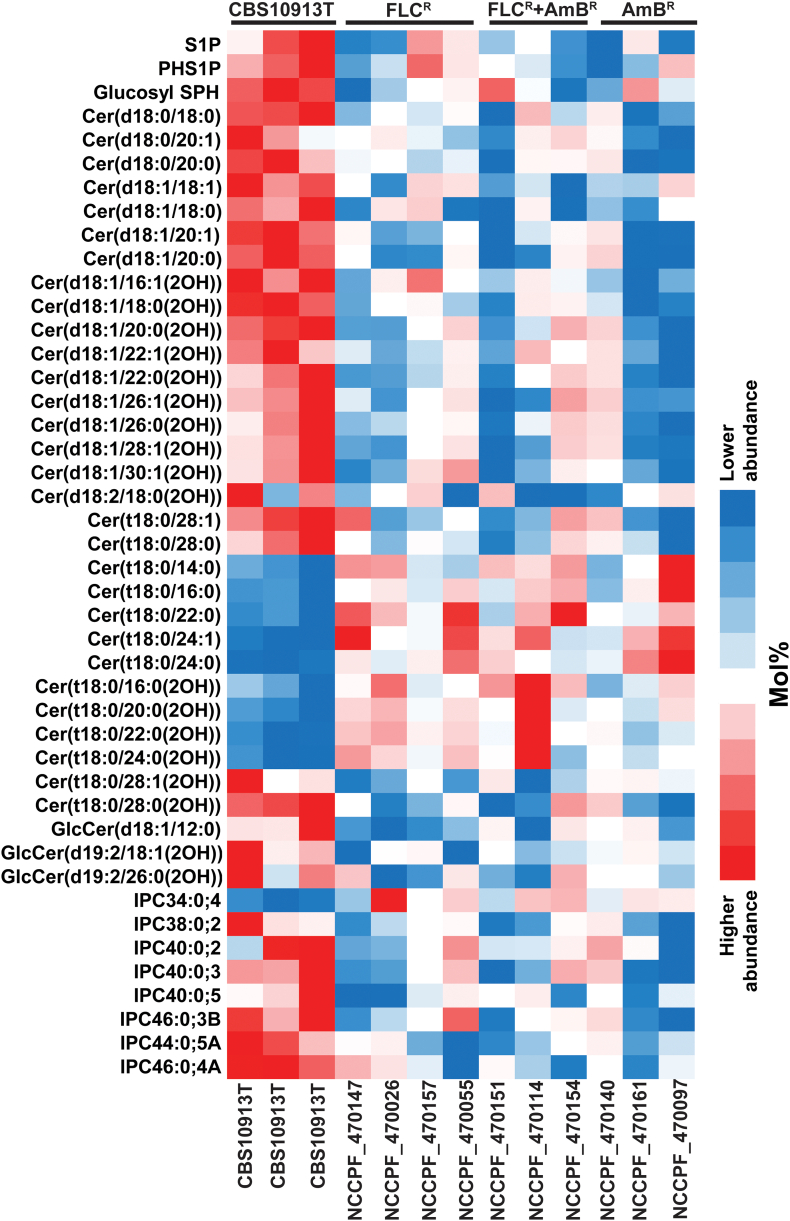
Comparative SLs profiling of susceptible (CBS10913T) and resistant isolates: The SLs species which display statistical significance (*p*-value ≤0.05) among CBS10913T and other three tested groups of isolates are depicted. 44 species from all nine groups of SLs show variation between susceptible and resistant isolates. The average value of 3 independent samples of each isolate is included except in the case of CBS10913T. Red and blue colors indicate higher and lower abundance of SLs, respectively.

We extended our analysis to include a drug susceptible *C. auris* CBS10913T isolate that further validated the fact that alterations in SLs are linked to drug susceptibilities (Table 1). A comparison between the SL profile of CBS10913T and various drug-resistant isolates highlighted differences at the molecular species-level wherein 44 SL species showed significant changes (Fig. 7, Supplementary file 1). Specifically, the dhCer, Cer, αOH-Cer and IPC species structures were abundant in CBS10913T strain as compared to the drug-resistant groups. On the other hand, PCer and αOH-PCer structures were present in low abundance in CBS10913T strain in comparison with the drug-resistant groups (Supplementary file 1). The comparative analysis between the drug susceptible CBS10913T and the drug resistant strains showed that specific SLs could be linked to drug resistance in *C. auris*. For example, the drug resistant strains show an accumulation of PCer and αOH-PCer at the expense of complex IPC structures. Such alterations could result from one of the two possible ways: (i) the IPC biosynthetic pathways slow down leading to an accumulation of precursors PCer and αOH-PCer; or (ii) upregulation of IPC degradation pathway (via ISC1) leading to an accumulation of PCer and αOH-PCer structures. In either scenario, the altered levels of PCer in membranes can alter the overall homeostasis affecting cellular functions in pathogenic fungi. Specific structural features like hydroxylated backbones and fatty acyls, are important for proper surface localization and organization of membrane proteins. For example, in *C. neoformans*, it was earlier reported that oligomerization of Pma1 (a plasma membrane ATPase) is severely affected in conditions where PCer levels are low [45]. Likewise, alterations in SLs could also influence the activity and/or localization of drug efflux pumps in plasma membrane, which in turn could affect the drug susceptibilities of *C. auris* [7,10,18]. However, further experimentation will be required to validate this hypothesis.

**Fig. 8:**
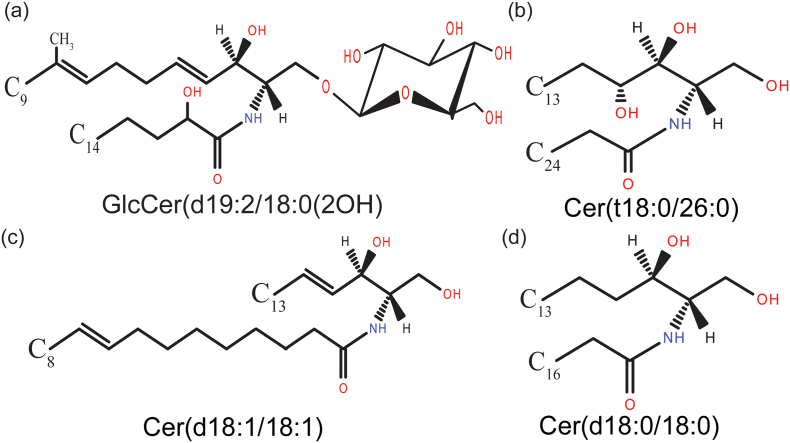
The structures depicted correspond to major SL species of *C. auris*. These lipid species are common in susceptible and drug-resistant isolates, only their contents vary between the isolates: (a) major glucosyl ceramide species (b) major phytoceramide species (c) major ceramide species (d) major dihydroceramide species.

Overall, our analyses revealed the presence of all major sphingoid bases in *C. auris*. Additionally, the study shows that some of the most abundant and distinguished sphingolipid structures present in *C. auris* include Cer(d18:1/18:1) and Cer(d18:0/18:0). While Cer(t18:0/26:0) is the major PCer structure, which represents the most abundant class of sphingolipids in *C. auris*, high levels of GlcCer with d19:2 glucosylceramide backbone were detected in AmB resistant *C. auris* isolates (Fig. 8). This study also highlighted the distinct molecular imprint among drug-resistant isolates. The significance of characteristics sphingoid bases molecular species in isolates resistance to different antifungals certainly deserves a closer look. This first sphingolipidomics fingerprint of *C. auris* should inform analyses of studies in drug resistance and virulence in this a newly emerging pathogen. Understanding the roles of individual genes of the identified SL biosynthetic pathway in the emerging drug resistance is poised to enlighten their relevance further.

The following are the supplementary data related to this article.

Supplementary file 1 The sphingolipid profiling data of various clinical isolates of C. auris. Data is represented in mol% (% of normalized total SL mass spectral signal); F: FLCR, FA: FLCR + AmBR, A: AmBR, CBS: CBS10913T; * represents species and classes that differ in at least 2 groups, ** represents species and classes that differ in at least 3 groups. Supplementary file 1

Supplementary file 2 Loading values of principal components 1, 2 and 3. The 12 highest and 12 lowest values are indicated. Supplementary file 2

Supplementary file 3 % Identity of C. auris SLs protein sequences with *C. albicans* and *S. cerevisiae* SLs protein sequences by protein BLASTp search. Supplementary file 3

Supplementary Table 1 The abundant SLs species of nine SLs classes that are found in the drug susceptible and all three sets of resistant isolates of *C. auris*. Average ± SEM of all classes is shown. *Represents the SL species which show statistically significant variation (*p* ≤ 0.05) between CBS10913T and all the other three groups of isolates. The complete dataset can be found in Supplementary file 1. Supplementary Table 1

## Data availability

All the datasets can be accessed either in the main manuscript or in supplementary files and access to all other primary data sets is available upon request.

## CRediT authorship contribution statement

**Mohit Kumar:** Conceptualization, Methodology, Investigation, Software, Data curation, Writing - original draft, Formal analysis, Validation, Writing - review & editing. **Ashutosh Singh:** Conceptualization, Methodology, Data curation, Writing - original draft, Formal analysis, Writing - review & editing, Funding acquisition. **Sonam Kumari:** Data curation, Investigation, Methodology, Writing - review & editing. **Praveen Kumar:** Data curation, Formal analysis. **Mohd. Wasi:** Data curation, Formal analysis. **Alok K. Mondal:** Supervision, Resources. **Shivaprakash M. Rudramurthy:** Data curation, Writing - review & editing. **Arunaloke Chakrabarti:** Data curation, Writing - review & editing. **Naseem A. Gaur:** Conceptualization, Resources, Writing - original draft, Writing - review & editing, Project administration, Funding acquisition, Supervision. **Neil A.R. Gow:** Supervision, Funding acquisition, Writing - review & editing, Project administration. **Rajendra Prasad:** Conceptualization, Resources, Writing - original draft, Writing - review & editing, Project administration, Funding acquisition, Supervision.

## Declaration of competing interest

The authors declare that there is no conflict of interest.
