# Supplementary material for: Sphingolipidomics of drug resistant Candida auris clinical isolates reveal distinct sphingolipid species signatures
Source: Biochim Biophys Acta Mol Cell Biol Lipids. 2021 Jan;1866(1):158815. doi: 10.1016/j.bbalip.2020.158815 (PMC7695621; doi:10.1016/j.bbalip.2020.158815)
Supplement: Supplementary Table 1 — The abundant SLs species of nine SLs classes that are found in the drug susceptible and all three sets of resistant isolates of C. auris. Average ± SEM of all classes is shown. *Represents the SL species which show statistically significant variation (p ≤ 0.05) between CBS10913T and all the other three groups of isolates. The complete dataset can be found in Supplementary file 1. [file mmc5.docx]

**Supplementary Table 1. The abundant SLs species of nine SLs classes that are found in the drug susceptible and all three sets of resistant isolates of *C. auris.*** Average ± SEM of all classes is shown. *Represents the SL species which show statistically significant variation (p ≤ 0.05) between CBS10913T and all the other three groups of isolates. The complete dataset can be found in supplementary file 1.

| **SL classes** | **SL species** | **SL content in the samples (Mole%)** | | | |
| --- | --- | --- | --- | --- | --- |
|  |  | **CBS10913T** | **FLC^R^** | **FLC^R^ + AmB^R^** | **AmB^R^** |
| Sphingoid base | SPH* | 0.014 ± 0.006 | 0.023 ± 0.005 | 0.013 ± 0.003 | 0.010 ± 0.002 |
|  | DHS | 0.265 ± 0.146 | 0.208 ± 0.03 | 0.134 ± 0.017 | 0.079 ± 0.009 |
|  | DHS1P | 0.086 ± 0.017 | 0.102 ± 0.014 | 0.089 ± 0.013 | 0.124 ± 0.031 |
|  | PHS | 0.225 ± 0.022 | 0.182 ± 0.023 | 0.197 ± 0.016 | 0.178 ± 0.027 |
|  | PHS1P* | 0.019 ± 0.003 | 0.011 ± 0.002 | 0.008 ± 0.001 | 0.008 ± 0.002 |
| dhCer | Cer(d18:0/18:0)* | 13.559 ± 0.351 | 9.787 ± 0.357 | 8.665 ± 1.01 | 7.784 ± 0.979 |
|  | Cer(d18:0/24:0) | 2.945 ± 0.673 | 9.537 ± 0.453 | 7.567 ± 1.32 | 5.846 ± 1.406 |
|  | Cer(d18:0/26:0) | 6.250 ± 1.835 | 4.811 ± 0.17 | 3.780 ± 0.710 | 2.940 ± 1.0 |
| Cer | Cer(d18:1/18:1)* | 0.342 ± 0.037 | 0.189 ± 0.014 | 0.151 ± 0.008 | 0.184 ± 0.015 |
|  | Cer(d18:1/18:0)* | 0.332 ± 0.049 | 0.165 ± 0.015 | 0.138 ± 0.15 | 0.148 ± 0.012 |
| αOH-Cer | Cer(d18:1/16:0(2OH)) | 0.469 ± 0.038 | 0.268 ± 0.029 | 0.316 ± 0.05 | 0.163 ± 0.025 |
|  | Cer(d18:1/18:0(2OH))* | 1.463 ± 0.153 | 0.235 ± 0.023 | 0.278 ± 0.043 | 0.147 ± 0.023 |
|  | Cer(d18:1/24:0(2OH)) | 0.829 ± 0.195 | 0.856 ± 0.068 | 0.847 ± 0.16 | 0.409 ± 0.114 |
|  | Cer(d18:1/26:0(2OH))* | 0.956 ± 0.328 | 0.279 ± 0.023 | 0.311 ± 0.075 | 0.207 ± 0.070 |
| PCer | Cer(t18:0/24:0)* | 5.665 ± 0.253 | 21.307 ± 1.141 | 19.382 ± 0.98 | 25.497 ± 2.329 |
|  | Cer(t18:0/26:0) | 18.573 ± 2.95 | 18.244 ± 0.0828 | 15.705 ± 0.824 | 14.641 ± 1.2 |
|  | Cer(t18:0/28:0)* | 7.129 ± 1.915 | 2.285 ± 0.153 | 2.273 ± 0.431 | 2.047 ± 0.352 |
| αOH-PCer | Cer(t18:0/24:0(2OH))* | 1.910 ± 0.247 | 5.190 ± 0.246 | 5.151 ± 0.636 | 4.423 ± 0.191 |
| GlcCer | GlcCer(d18:1/16:0) | 1.227 ± 0.334 | 1.243 ± 0.16 | 1.237 ± 0.202 | 0.781 ± 0.113 |
|  | GlcCer(d18:1/18:1) | 0.970 ±0.281 | 0.737 ± 0.010 | 0.778 ± 0.092 | 0.844 ± 0.047 |
| αOH-GlcCer | GlcCer(d19:2/18:0(2OH) | 24.488 ± 6.336 | 13.343 ± 1.606 | 21.993 ± 4.011 | 23.722 ± 2.45 |
| IPC | IPC42:0;4B | 0.063 ± 0.024 | 0.122± 0.007 | 0.097 ± 0.014 | 0.148 ± 0.029 |
|  | IPC46:0;4B | 0.070 ± 0.019 | 0.134± 0.035 | 0.129 ± 0.024 | 0.058 ± 0.015 |
